# Influence of Population Immunosuppression and Past Vaccination on Smallpox Reemergence

**DOI:** 10.3201/eid2404.171233

**Published:** 2018-04

**Authors:** C. Raina MacIntyre, Valentina Costantino, Xin Chen, Eva Segelov, Abrar Ahmad Chughtai, Anthony Kelleher, Mohana Kunasekaran, John Michael Lane

**Affiliations:** School of Public Health and Community Medicine, University of New South Wales, Sydney, New South Wales, Australia (C.R. MacIntyre, V. Costantino, X. Chen, A.A. Chughtai, M. Kunasekaran);; Arizona State University, Phoenix, Arizona, USA (C.R. MacIntyre);; Monash University and Monash Health, Melbourne, Victoria, Australia (E. Segelov);; Kirby Institute, University of New South Wales, Sydney (A. Kelleher);; Emory University, Atlanta, Georgia, USA (J.M. Lane)

**Keywords:** smallpox, modeling, immunocompromised, residual immunity, viruses, HIV/AIDS, bioterrorism and preparedness, Australia, United States, New York City, Sydney, vaccination, vaccinia, New York, immunosuppression, poxviruses

## Abstract

We built a SEIR (susceptible, exposed, infected, recovered) model of smallpox transmission for New York, New York, USA, and Sydney, New South Wales, Australia, that accounted for age-specific population immunosuppression and residual vaccine immunity and conducted sensitivity analyses to estimate the effect these parameters might have on smallpox reemergence. At least 19% of New York’s and 17% of Sydney’s population are immunosuppressed. The highest smallpox infection rates were in persons 0–19 years of age, but the highest death rates were in those >45 years of age. Because of the low level of residual vaccine immunity, immunosuppression was more influential than vaccination on death and infection rates in our model. Despite widespread smallpox vaccination until 1980 in New York, smallpox outbreak severity appeared worse in New York than in Sydney. Immunosuppression is highly prevalent and should be considered in future smallpox outbreak models because excluding this factor probably underestimates death and infection rates.

Smallpox virus was eradicated in 1980 but remains a category A bioterrorism agent (*1*). The only official stocks of the virus are in the United States and Russia (*2*), but unofficial stocks could be present elsewhere. Advances in synthetic biology of poxviruses and availability of the full variola genome sequence make synthesis of smallpox virus in the laboratory possible (*3*). Smallpox could reemerge as a result of bioterrorism or a laboratory accident (*4*); thus, smallpox is a high priority for preparedness planning (*5*). Given that smallpox is eradicated, mathematical models enable researchers to predict the effects of a smallpox epidemic, but these predictions depend critically on the assumptions of the mathematical model.

Many researchers who have developed smallpox models have been optimistic about residual vaccine-induced immunity and assumed a case-fatality ratio (CFR) of 30%, whereas estimates of outbreaks in nonimmune populations suggest a CFR of 50%–70% (*6*). Given the absence of smallpox in the world for nearly 40 years and loss of immunologic boosting from wild-type infection, the CFR of an epidemic today might be higher.

The immunologic status of the population has also changed dramatically in the decades since smallpox eradication. A larger proportion of the population today is unvaccinated, and residual immunity in persons who were vaccinated before 1980 is waning (*7*). In addition, the prevalence of HIV, advances in transplantation, and therapies for cancer and many autoimmune conditions have resulted in unprecedented rates of immunosuppression (*8*). In 1980, when smallpox was eradicated, HIV had not yet manifested a high global burden of disease. Similarly, bone marrow transplantation was in its infancy, and heart–lung transplantations had not yet occurred. The fact that the proportion of unvaccinated and immunosuppressed persons in the population is increasing has not yet been adequately considered in estimations of the effect of reemergent smallpox.

Persons born after 1980 have no immunity to smallpox because they have never been exposed to wild-type virus or been vaccinated. For vaccinated cohorts, immunity wanes over time, and the highest protection is present during the first 5 years after vaccination, possibly waning to zero within 5–10 years (*9*). Furthermore, immunosenescence is a predictable, exponential decline in immune function that occurs after 50 years of age (*10*) and reduces the body’s ability to fight infection and respond to vaccines (*11*). This phenomenon further adds to immunosuppression in countries with an aging population. The aim of this study was to estimate the effect of reemerging smallpox in New York, New York, USA, and Sydney, New South Wales, Australia, 2 large cities with different vaccination histories for which estimates could be made on the population’s immunologic status.

## Methods

### Population

**Figure 1:**
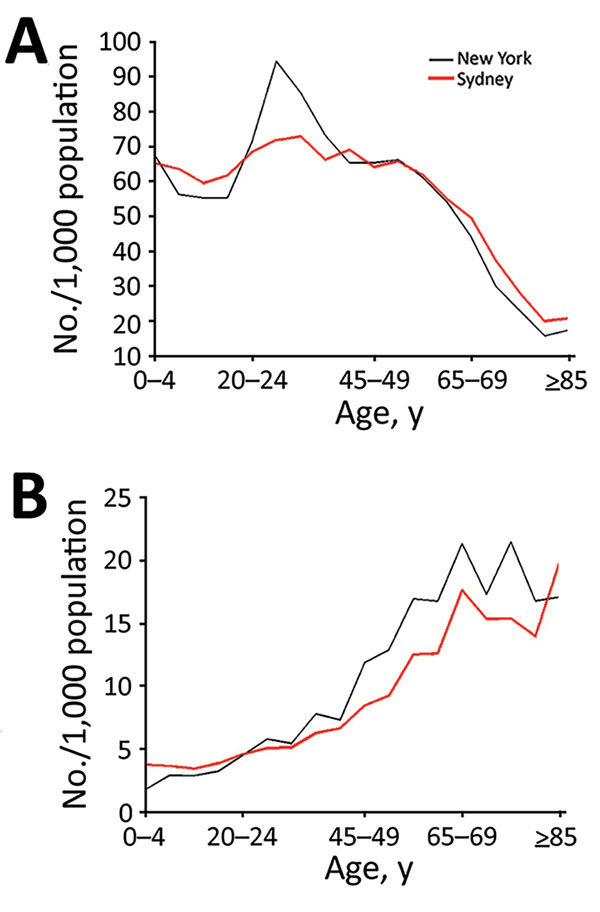
Characteristics of population used to model smallpox transmission, by age group, New York, NY, USA, and Sydney, New South Wales, Australia. Characteristics (e.g., size, age, immunosuppression rates) of populations from 2015 were used. A) Total population; B) immunosuppressed population.

We used Sydney’s population in 2015 (*12*), which was estimated using data from the state of New South Wales (*13*). The New York population of the same year was derived from the relevant statistical collection (*14*). We divided both populations into 5-year age groups up through ages 80–84 years and combined the eldest (persons >84 years of age) into a single group (Figure 1, panel A). Each age group was divided into vaccinated and unvaccinated compartments, which were then further subdivided into 3 categories of immunity: immunocompetent, mildly immunosuppressed, and moderate-to-severely immunosuppressed. We assumed that immunosuppressed persons had no residual immunity from vaccination.

### Immunosuppressed Population

We considered common types of immunosuppression estimated in an influenza study (*15*). We classified persons into 2 categories of immunosuppression: moderate to severe (called severe in our model) and mild. Severe immunosuppression was defined as a condition in which quantifiable data existed to demonstrate a risk for infection more than twice that of an immunocompetent person. This classification was left as a single category in the absence of reliable methodology to subdivide it. Mild immunosuppression was defined as a condition in which immunosuppression was documented but susceptibility to infection was estimated to be less than twice that of an immunocompetent host. For the analysis, persons with severe immunosuppression were assumed to have 2× and mild immunosuppression 1.5× the susceptibility to infection of a healthy person (*16*).

We sourced data for each city, and when only countrywide data were available, we attributed rates from the countrywide data set to the respective fraction of the population in the city. When age-specific immunosuppression prevalence data were not available, we used yearly age-specific incidence data instead to calculate prevalence age distribution (*17*,*18*).

We estimated the populations living with cancer (*17*,*19*), HIV (*20*,*21*), organ transplants (*22*,*23*), respiratory syndromes such as asthma (*24*,*25*) and chronic obstructive pulmonary disease (*26*,*27*), dialysis (*28*,*29*), and autoimmune diseases (*30*,*31*) and divided these populations into the 2 immunosuppression categories for each city (Technical Appendix Table 1). We acknowledge that many other diseases are associated with immunosuppression. Our method underestimates the amount of immunosuppression in the population.

### Vaccine-Induced Residual Immunity

In the United States, including New York, widespread smallpox vaccination occurred until 1970 (*32*). In contrast, in the geographically isolated island continent of Australia, quarantine was used to protect the population because smallpox was never endemic (*32*). Widespread vaccination never occurred in Australia; only the armed forces and healthcare workers were vaccinated, which occurred until 1979, although reactive vaccination campaigns had been conducted during a smallpox outbreak in Sydney in 1917 (*33*).

For New York, we assumed 80% of the healthy population 40–69 years of age (born before 1975) were previously vaccinated. For Sydney, we estimated the proportion of persons vaccinated by estimating those born before 1980 in the following groups: healthcare workers in Sydney in 2015 (*34*), members of the defense forces, and migrants (>30% in the Sydney population) (*35*), who might have been vaccinated in their country of origin (≈80,000 persons). We estimated that, in Sydney, at most 30% of the total population born before 1980 (persons 35–69 years of age) had been vaccinated. On the basis of a mathematical model (*36*) that estimated waning immunity against severe smallpox as 1.41% per year after vaccination, we calculated the age-specific residual protection for vaccinated persons 40–69 years of age by multiplying that percentage by the number of years from vaccination and subtracting it from 100% starting protection.

### Contact Mixing

In our model, we used the heterogeneous age-specific contact rates from the European mixing patterns study (*37*). We assumed that persons would greatly reduce their social contacts after becoming symptomatic with smallpox (*38*). To account for this change in social contact, we modified the normal contact matrix, multiplying the matrix by a factor (0 < *x* < 1) to reduce the number of contacts per day (*39*). Because of the lack of studies quantifying this reduction, we assumed *x* to be 0.5, as in a previous study (*39*). Considering severe smallpox types are more substantially prostrating, we applied the reduced contact matrix to hemorrhagic and flat smallpox infections from the first day of illness. For ordinary smallpox, we assumed the behavior change started on the second day and for vaccine-modified smallpox, on the third day.

### Disease Types

We categorized smallpox disease into 4 different types defined by infectivity (R_0_) and CFRs: hemorrhagic, flat, ordinary, and vaccine-modified. Age-specific and other model parameters (Technical Appendix Table 2) as well as further model details are explained in the Technical Appendix.

### Smallpox Disease Type Distribution

We assumed infected persons had different probabilities of developing each disease type, depending on their age and immunologic status. The incidence of each disease type within each age group for healthy unvaccinated persons was drawn from historical outbreaks (*9*) (Technical Appendix Table 3). For healthy unvaccinated persons, hemorrhagic smallpox ratios ranged from 7 cases/1,000 persons infected (in the 5–9-year age group) to 200 cases/1,000 persons infected (in the >85-year age group). Flat smallpox age-specific rates were lowest for the 10–14-year age group (30 cases/1,000 persons infected) and reached 180 cases/1,000 persons infected for the oldest age group. For the mildly immunocompromised population, we doubled the age-specific probability of hemorrhagic and flat smallpox. We assumed 100% of severely immunocompromised persons would develop hemorrhagic disease. We assumed the vaccinated subgroup had reduced susceptibility and rates of severe smallpox types. We estimated that 25.3% of vaccinated persons would get vaccine-modified smallpox (*9*). We applied a waning immunity function over time at a rate of 1.41% per year from vaccination (*36*) and assumed the rates of hemorrhagic and flat smallpox would increase with time from vaccination while rates of vaccine-modified smallpox would decrease with time from vaccination (Technical Appendix Table 4).

### Mathematical Model

We constructed a modified SEIR (susceptible, exposed, infected, recovered) model for smallpox transmission (Technical Appendix Table 2). The population was divided into vaccinated and unvaccinated compartments, which were then further subdivided into 3 categories of immunity: immunocompetent, mildly immunosuppressed, and moderate-to-severely immunosuppressed. The model used ordinary differential equations to move populations into epidemiologic states related to their smallpox infectious status: susceptible, infected, prodromal, infectious, recovered, or dead. Once infected, populations were moved into the next state on the basis of disease duration rates. To obtain the age-specific force of infection (i.e., the rate at which susceptible persons acquire smallpox), we used the Euler approximation to make discrete contact rates, assuming the rates were proportional to the patterns observed in the United Kingdom. Then, to account for the different infectivity rates of different smallpox types, we estimated the transmission parameter β (i.e., the probability of getting infected from a contact) for each smallpox disease type to calculate the R_0_ for hemorrhagic, flat, ordinary, and vaccine-modified smallpox. Finally, we multiplied the force of infection by a parameter (α_1,_ α_2,_ α_3,_ α_4_; Technical Appendix Table 2) to account for the different susceptibility levels of different populations.

The model ran for 100 simulated days. We assumed an attack in a crowded public space, such as an airport, and started the epidemic with 51 infected in New York and 29 in Sydney to reflect the same attack rate for each population. We assumed a dynamic population updated each day using the birth (*40*) and age-specific death rates (*41*,*42*) from 2014 for both cities.

### Sensitivity Analysis

We conducted a sensitivity analysis on the assumption of waning immunity, reducing immunity by 0.7% per year (approximately half the value used in the base case scenario [i.e., the first scenario discussed]). We present results for 3 different assumptions about residual vaccine immunity: no residual immunity, base case immunity (1.41% waning immunity per year), and high residual immunity (0.7% waning immunity per year). We also conducted a sensitivity analysis to test the model outputs without considering population immunosuppression, which has been the approach in most past models (*43*).

## Results

### Population and Immunity Levels

We examined the population age distributions of New York and Sydney. Sydney has a higher percentage of persons <20 and >55 years of age than New York (Figure 1, panel A), whereas New York has a higher proportion of persons 20–39 years of age than Sydney. We estimated that 4.54% of New York’s population and 3.76% of Sydney’s population are severely immunocompromised, 14.81% of New York’s population and 12.95% of Sydney’s population are mildly immunocompromised, 59.14% of New York’s population and 72.56% of Sydney’s population are healthy unvaccinated, and 21.51% of New York’s population and 10.73% of Sydney’s population are healthy vaccinated. Similar proportions of the 2 cities’ populations (19% in New York and 17% in Sydney) are immunosuppressed (Figure 1, panel B). New York has a higher proportion of the population vaccinated (21%) than Sydney (10%).

### Base Case Scenario

**Figure 2:**
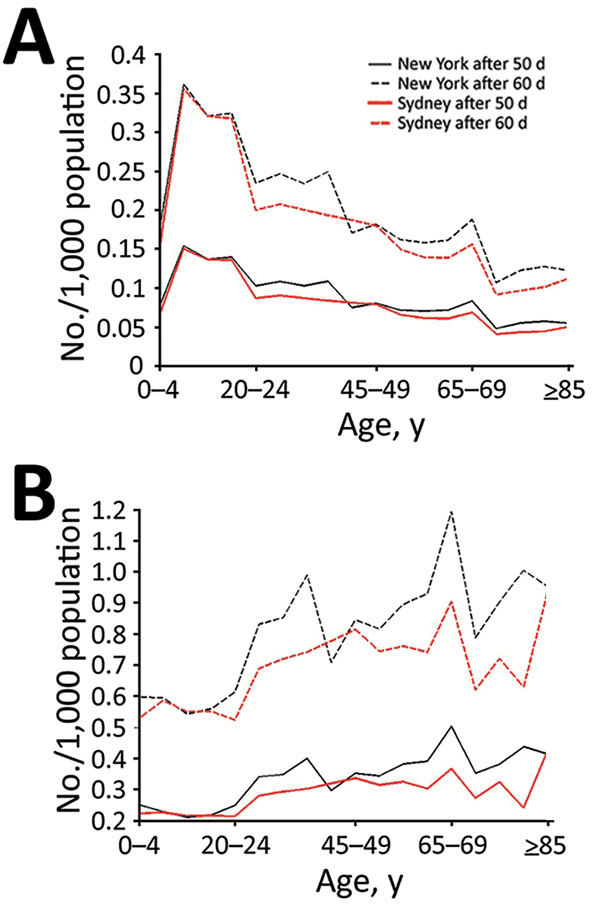
Smallpox infection and death rates of population for base case scenario and for scenario including immunosuppression in model, by age group, New York, NY, USA, and Sydney, New South Wales, Australia. Characteristics (e.g., size, age, immunosuppression rates) of populations from 2015 were used. A) Infection rate 50 and 60 days after start of smallpox outbreak; B) cumulative deaths in population 50 and 60 days after start of smallpox outbreak.

We analyzed age-specific infection (Figure 2, panel A) and death (Figure 2, panel B) rates using the base case scenario (medium immunity level) including the immunosuppressed population. Persons 5–19 years of age are at highest risk for smallpox infection in both cities (Figure 2, panel A). Although the proportion of persons infected in both cities is similar among the 0–19-year age groups, ≈25% more persons in New York than Sydney become infected among the 20–39-year age groups.

Cumulative deaths per 1,000 population increase with age starting with persons >20 years of age (Figure 2, panel B). Deaths peak in the 65–69-year age group in both cities, reaching 1.2 deaths/1,000 population for New York and 0.9 deaths/1,000 population for Sydney 60 days after the start of the outbreak; rates increase again in those >80 years of age. The New York population also has a smaller peak in deaths in the 35–39-year age group. Although the spread of infection is mostly driven by higher contact rates among persons of young age groups, the peaks in death reflect the distribution of the immunosuppressed population (Figure 1, panel B; Figure 2, panel B). The effect of residual immunity is more apparent in New York trends, which show a greater decrease in infections and cumulative deaths in the age groups that were previously vaccinated (40–65 years of age).

**Figure 3:**
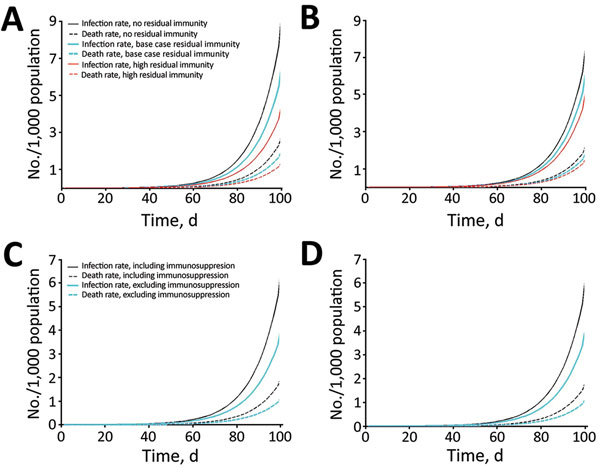
Smallpox infection and death rates over time considering different immunologic factors included in model, New York, NY, USA, and Sydney, Australia. Characteristics (e.g., size, age, immunosuppression rates) of populations from 2015 were used. A) Rates for New York, considering different levels (none, base case, and high) of residual vaccine immunity with the inclusion of immunosuppressed population. B) Rates for Sydney, considering different levels (none, base case, and high) of residual vaccine immunity with the inclusion of immunosuppressed population. C) Rates for New York, including and excluding immunosuppression with base case level of residual vaccine immunity. D) Rates for Sydney, including and excluding immunosuppression with base case level of residual vaccine immunity.

Looking at total rates over time, New York (Figure 3, panels A, C) and Sydney (Figure 3, panels B, D) have similar exponential growths of infection rates, with slightly higher trends for New York. The rate of infection reaches 0.094 infected/1,000 population for New York and 0.084 infected/1,000 population for Sydney 50 days after the smallpox introduction and increases to 0.496 infected/1,000 population for New York and 0.452 infected/1,000 population for Sydney by 70 days. The death rates are 0.028 deaths/1,000 population for New York and 0.025 deaths/1,000 population for Sydney after 50 days and reach 0.151 deaths/1,000 population for New York and 0.133 deaths/1,000 population for Sydney by 70 days.

### Residual Immunity Analysis

**Figure 4:**
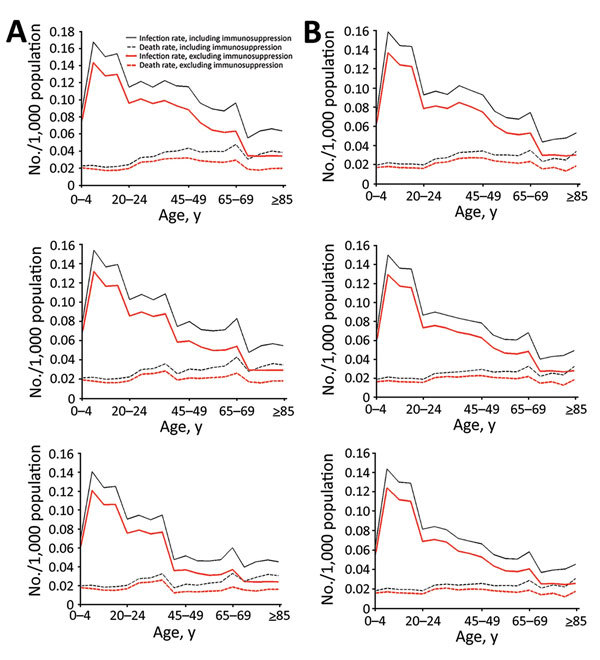
Smallpox infection and death rates with different levels of residual vaccine immunity including and excluding immunosuppression in model of smallpox transmission, by age group, New York, NY, USA, and Sydney, Australia. Characteristics (e.g., size, age, immunosuppression rates) of populations from 2015 were used. A) New York 50 days after start of smallpox outbreak with no (top), base case (middle), and high (bottom) residual vaccine immunity. B) Sydney 50 days after start of smallpox outbreak with no (top), base case (middle), and high (bottom) residual vaccine immunity.

Infection and death rate estimates for New York, where vaccine coverage is more than double that of Sydney, are more sensitive to assumptions of residual immunity. New York (Figure 3, panel A) has lower rates of infection than Sydney (Figure 3, panel B) only in the scenario of high residual immunity. At day 50 of the outbreak, rates are ≈15% (base case residual immunity) and 31% (high residual immunity) lower in New York and 10% (base case residual immunity) and 17% (high residual immunity) lower in Sydney with residual immunity than without residual immunity. Differences in infection and death rates among different residual immunity assumptions increased with time. Regarding the impact on age-specific rates in New York (Figure 4, panel A) and Sydney (Figure 4, panel B), the assumption of high residual immunity produced lower death rates for the older age groups.

### Immunosuppression Analysis

Infection and death rates increase when including (vs. excluding) immunosuppression parameters in the model; greater differences are seen between New York’s infection rates (Figure 3, panel C) and Sydney’s infection rates (Figure 3, panel D). The difference in rates increases with time, reaching ≈20% in New York and 18% in Sydney at day 50 from the start of the outbreak and 28% for New York and 25% for Sydney at day 70. Although including immunosuppression estimates into the model produced similarly higher infection rates for each age group (less for the 0–4-year age group), differences in death rates increased with age (Figure 4).

## Discussion

With each passing year, population immunosuppression is a more influential determinant than residual vaccine immunity of the severity of a smallpox epidemic. Although the spread of disease is highest in younger age groups, driven mostly by their higher contact rates, higher death rates were seen in older populations, reflecting the prevalence of immunosuppression.

The differences between New York, which has high vaccination coverage (an estimated ≈22% of the population), and Sydney, which has low (≈10%) vaccination coverage, demonstrate that residual immunity assumptions are not as influential in Sydney as in New York. However, the consideration of population immunosuppression, from medical conditions to iatrogenic factors, strongly affects disease transmission and deaths in both cities. This large population subset must be considered when modeling the impact of any infectious disease outbreak. We estimated conservatively that almost 1 in 5 persons in New York and 1 in 6 persons in Sydney (and higher for the 60–64-year age group) are living with some degree of immunosuppression. Although New York has higher rates of immunosuppression for the 25–84-year age groups, Sydney has higher rates than New York for the youngest (0–19 years) and the oldest (>85) populations.

Residual immunity affects age-specific infection and death rates, with both cities showing the highest infection rates for unvaccinated young persons 5–19 years of age. However, death rates rise after 40 years of age, despite higher vaccination coverage in this age group. For Sydney, even an assumption of higher immunity does not affect the infection or death rates greatly because of the low vaccine coverage before 1980. However, residual immunity becomes more influential if we use more optimistic assumptions of waning immunity. Note that persons who have been vaccinated would mount a more robust and rapid response to revaccination in the event of an outbreak and might be better protected after postexposure vaccination. Obtaining a vaccination history and checking for a consistent scar are necessary parts of outbreak management.

Although immunosuppression is a major determinant of the size and distribution of a smallpox outbreak, this fact should not be a major determinant of vaccination policy. Immunosuppression should continue to be an absolute contraindication for vaccination of persons who are not true contacts. Ensuring that persons with immunosuppression (including healthcare workers) avoid contact with persons with smallpox (if possible) should be a priority. Smallpox would always be more pathogenic than vaccinia virus, so any patient with a bona fide exposure to smallpox should be vaccinated with a fully potent vaccinia strain, such as ACAM2000 (*44*). If such patient develops a serious complication, such as eczema vaccinatum or progressive vaccinia, the patient can be treated with ST-246 (Siga Technologies, New York, NY, USA) (*45*).

Our study is subject to some limitations. We used an underestimate of immunosuppression; other conditions causing immunosuppression, such as diabetes, were not considered. We also used conservative estimates for the increased risk for infection in immunosuppressed persons and grouped persons with severe and moderate immunosuppression into single categories because of the absence of more specific data to categorize them further by degree of immunosuppression. The contact matrix we used was estimated in a study conducted in the United Kingdom in 2006, which might not necessarily reflect New York or Sydney social contact patterns (*37*). Furthermore, contacts with symptomatic infectious patients will probably drop to near zero once an outbreak has been confirmed and patients are well isolated, assuming adequate health system capacity for isolation and treatment of smallpox patients. The data in the model on age-specific rates of smallpox were obtained from hospitalized patients (*9*), which might overestimate the rates of severe disease in the model outputs.

The speed and vigor with which smallpox control efforts are implemented should be major aspects of control efforts and need to be tested in a model that accounts adequately for immunosuppression. Ensuring adequate hospital care and isolation facilities will also help in epidemic control. During the Ebola epidemic in West Africa, lack of beds resulted in widespread community transmission, and modeling showed that 70% of patients needed to be in treatment facilities to control the epidemic (*46*). The response to severe acute respiratory syndrome, with its rapid control despite the lack of a vaccine or antiviral agent, showed that patient isolation can be very successful (*47*). Experiences with severe acute respiratory syndrome, Ebola, and Middle East respiratory syncytial coronavirus also illustrate the heavy toll on healthcare workers (*48*), who should be assumed to be at high risk for infection in the event of a smallpox outbreak.

Given waning smallpox vaccine immunity (nearly 4 decades since eradication and a dwindling vaccinated population), the influence of population immunosuppression is greater than that of residual vaccine immunity, yet has not been adequately considered in smallpox epidemic modeling. Advances in medicine and new endemic diseases, such as HIV, have resulted in almost 1 in 5 persons living with immunosuppression in large metropolitan cities. Immunosuppression must be considered in preparedness planning and poses a challenge for vaccination strategies during potential smallpox outbreaks.

Technical Appendix Description of the SEIR (susceptible, exposed, infected, recovered) model of smallpox transmission and model parameters.
