# Supplementary material for: Influence of Population Immunosuppression and Past Vaccination on Smallpox Reemergence
Source: Emerg Infect Dis. 2018 Apr;24(4):646–53. doi: 10.3201/eid2404.171233 (PMC5875263; doi:10.3201/eid2404.171233)
Supplement: Technical Appendix — Description of the SEIR (susceptible, exposed, infected, recovered) model of smallpox transmission and model parameters. [file 17-1233-Techapp-s1.pdf]

Article DOI: https://doi.org/10.3201/eid2404.171233

Influence of Population Immunosuppression and Past Vaccination on Smallpox Reemergence

Technical Appendix

Estimation of Immunosuppressed Population

Sydney, Australia

The numbers of persons living with cancer in Australia were estimated to be 906,000 in 2010 and 1.1 million in 2015 (*1*), which represents 4.6% of the total population. We used age-specific cancer incidence data (*1*) to distribute this prevalence across ages. We estimated ≈15,700 persons were living with HIV in New South Wales (NSW), Australia, in 2015 (*2*). We estimated that 33,000 persons in Australia were living with transplants by 2015, which represents 0.14% of the total population. We distributed this estimated prevalence using the age distribution estimated in the United States, which was applied to both Sydney and New York, NY, USA. An estimated 11% of persons living in Australia have asthma, and 5.2% of persons ≥45 years have chronic obstructive pulmonary disease (COPD) (*3*). At the end of 2014, a total of 12,091 persons in Australia, 3,872 being in NSW, were receiving dialysis (*4*). We divided this population up following age-specific incidence estimates (*4*) and adjusted for the Sydney population. Finally, we included the proportion of persons living with autoimmune diseases, estimated to be 5% in Australia (*5*), and distributed this number through age groups following the prevalence distribution of rheumatoid arthritis, one of the most common autoimmune diseases.

New York

In New York, the HIV prevalence has been estimated at 0.37% of the total population (*6*). Persons living with cancer were estimated to be 4.78% in 2010 (*7*); we divided up the estimates of persons living with HIV or cancer by age group accordingly to age-specific incidence statistics (*7*). About 500,000 transplants were performed in the United States during 1987–2015 (*8*), representing 0.15% of the total population. Asthma and COPD were estimated to affect 7.7%

(*9*) and 7.84% (*10*) of the population, respectively. Persons living with autoimmune diseases and dialysis were estimated to be 7.37% (*11*) and 0.14% (*12*), respectively. We distributed dialysis treatment prevalence by age (*6*) and autoimmune diseases by rheumatoid arthritis age-specific incidence.

Division of Estimated Immunosuppressed Population into Different Immunologic Levels

At any point in time, a fraction of cancer patients receiving chemotherapy will be severely immunosuppressed; we estimated this fraction to be 6.7% of persons with cancer (*1*). We classified this fraction as severely immunosuppressed and the remaining 93.3% as mild.

We considered 30% of the persons in Sydney and 40% of the persons in New York living with HIV, 100% of persons living with organ transplant, and 33% of the population effected by autoimmune diseases to be severely immunocompromised (Technical Appendix Table 1). The mildly immunocompromised compartment comprised the rest of the population living with HIV (70%–60%), autoimmune diseases (67%), cancer (97.3%), and renal dialysis (100%). Use of inhaled corticosteroids is immunosuppressive to the respiratory tract and associated with a higher incidence of pneumonia. We divided the population affected by COPD and asthma into the severe (12.3%) and mildly immunocompromised (39.2%) and considered the rest (48.5%) not immunosuppressed.

Disease Type

Persons infected with hemorrhagic and flat smallpox have the highest basic reproduction number estimated from different historical outbreaks ($R_0 = 10$) (*13*); however, we used an R_0 of 5 to account for the isolation of severely ill patients (Technical Appendix Table 2). For ordinary smallpox, we assumed an R_0 of 7.96, estimated from a detailed study of an outbreak in Nigeria in a unvaccinated community (*14*), and for modified smallpox, we assumed an R_0 of 5.3 (2/3 of the R_0 estimated for the ordinary type). Because of milder symptoms, we accounted for isolation and halved R_0 from the third and fourth day for ordinary and modified smallpox, respectively. Data collected from historical outbreaks (*15*) shows that persons infected with hemorrhagic, flat, and vaccine-modified smallpox have 100%, 90%, and 0% case fatality ratios (CFRs), respectively, while for ordinary smallpox, infection is age-specific (*15*). Another study showed the CFR for hemorrhagic type being 95% (*16*). In our study, due to better outbreak response and more developed care centers, we assumed the same CFR for ordinary and vaccine-

modified smallpox, but a slightly lower CFR for hemorrhagic and flat cases, being 90% and 75%, respectively.

Model Parameters

All the parameters used in the SEIR model are listed in Technical Appendix Table 2.

Age Distribution Rates of Each Smallpox Disease Type

We used age-specific rates for hemorrhagic, flat, and ordinary smallpox as estimated by Rao (*15*). To get age-specific rates for all age groups up to the ≥85-year age group, we linearly interpolated the values for the age group available from data collected. The age-specific rates used in the model for each age group of the healthy unvaccinated population are shown in Technical Appendix Table 3.

For the mildly immunosuppressed population, we doubled the age-specific rates of severe smallpox types (Technical Appendix Table 2), while for the severely immunosuppressed population, we set at 100% because only the hemorrhagic type develops. Regarding the vaccinated subgroup of the population, an estimated 25.3% of vaccinated persons get vaccine-modified smallpox (*15*). We applied a waning immunity function over time at a rate of 1.41% per year after vaccination (*17*). We applied the same waning for the susceptibility as well, starting with a 100%-effective vaccination. Age-specific rates for this subgroup are shown in Technical Appendix Table 4.

References

1. Australian Government. Cancer in Australia statistics. 2017 [cited 2017 Feb 15]. https://canceraustralia.gov.au/affected-cancer/what-cancer/cancer-australia-statistics

2. New South Wales Ministry of Health. NSW HIV strategy 2012 – 2015. 2015 [cited 2017 Feb 15]. http://www.health.nsw.gov.au/endinghiv/Documents/q4-2015-and-annual-hiv-data-report.pdf

3. Australian Institute of Health and Welfare. Chronic diseases data. 2016 [cited 2018 Feb 8]. https://www.aihw.gov.au/reports/life-expectancy-death/grim-books/contents/grim-books#page2

4. McDonald S, Chang S, Excell L, editors. Australia and New Zealand dialysis and transplant registry. Adelaide, South Australia: The Queen Elizabeth Hospital; 2008. http://www.anzdata.org.au/anzdata/AnzdataReport/31stReport/ContentsandSummary.pdf

5. Australasian Society of Clinical Immunology and Allergy. Autoimmune diseases. 2017 [cited 2017 Mar 24]. https://www.allergy.org.au/patients/autoimmunity/autoimmune-diseases

6. Centers for Disease Control and Prevention. Diagnoses of HIV infection in the United States and dependent areas, 2015. HIV Surv Rep. 2015;27. [cited 2017 Mar 22]. https://www.cdc.gov/hiv/pdf/library/reports/surveillance/cdc-hiv-surveillance-report-2015-vol-27.pdf

7. New York State, Department of Health. NYS cancer registry and cancer statistics. 2017 [cited 2018 Feb 8]. https://www.health.ny.gov/statistics/cancer/registry/

8. United Network for Organ Sharing. Annual reports. A look back at UNOS' achievements. 2015 [cited 2017 Mar 16]. https://www.unos.org/about/annual-report/

9. Centers for Disease Control and Prevention. Most recent asthma data. 2017 [cited 2017 Mar 16]. https://www.cdc.gov/asthma/most_recent_data.htm

10. Fenner F, Henderson DA, Arita I, Ježek Z, Ladnyi ID. Smallpox and its eradication. Geneva: World Health Organization; 1987.

11. Cooper GS, Bynum ML, Somers EC. Recent insights in the epidemiology of autoimmune diseases: improved prevalence estimates and understanding of clustering of diseases. J Autoimmun. 2009;33:197–207. PubMed http://dx.doi.org/10.1016/j.jaut.2009.09.008

12. World Health Organization. Smallpox [cited 2017 Feb 28]. http://www.who.int/csr/disease/smallpox/en/

13. Eichner M, Dietz K. Transmission potential of smallpox: estimates based on detailed data from an outbreak. Am J Epidemiol. 2003;158:110–7. PubMed http://dx.doi.org/10.1093/aje/kwg103

14. Stockdale JE, Kypraios T, O'Neill PD. Modelling and Bayesian analysis of the Abakaliki smallpox data. Epidemics. 2017;19:13–23. PubMed

15. Rao AR. Smallpox. Bombay, India: Kothari Book Depot; 1972.

16. US Army Medical Research Institute for Infectious Disease. USAMRIID's medical management of biological casualties handbook. 4th ed. Editors Kortepeter M, Christopher GW, Cieslak T, Culpepper R, Darling R, Pavlin J, et al. Frederick (MD): Department of Defense, Army Medical Department, Army Medical Research Institute for Infectious Disease; 2001.

17. Nishiura H, Eichner M. Infectiousness of smallpox relative to disease age: estimates based on transmission network and incubation period. Epidemiol Infect. 2007;135:1145–50. PubMed http://dx.doi.org/10.1017/S0950268806007618

18. Australian Bureau of Statistics. 3105.0.65.001 - Australian historical population statistics, 2014. 2014 Sep 18 [cited 2017 Mar 14]. http://www.abs.gov.au/AUSSTATS/abs@.nsf/mf/3105.0.65.001

19. Koblentz GD. The de novo synthesis of horsepox virus: implications for biosecurity and recommendations for preventing the reemergence of smallpox. Health Secur. 2017;15:620–8. PubMed http://dx.doi.org/10.1089/hs.2017.0061

Technical Appendix Table 1. Estimation of immunosuppressed population, by disease and immunosuppression level, New York, NY, USA, and Sydney, Australia

Disease	Severely immunosuppressed, %	Mildly immunosuppressed, %	Healthy, %
HIV	Sydney 30; New York 40	Sydney 70; New York 60	0
Organ transplants	100	0	0
Cancer	6.7	93.3	0
Asthma	12.3	39.3	48.5
Chronic obstructive pulmonary disease	12.3	39.3	48.5
Autoimmune diseases	33	67	0
Renal dialysis	0	100	0

Technical Appendix Table 2. Parameters used in SEIR model for smallpox transmission, New York, NY, USA, and Sydney, Australia*

Symbol	Definition	Value
α_1	Susceptibility of severely immunocompromised (*18*)	2
α_2	Susceptibility of mildly immunocompromised (*18*)	1.5
α_3	Susceptibility of healthy unvaccinated persons	1
α_4	Susceptibility and reduced probability of hemorrhagic and flat smallpox for vaccinated persons 35–69 years of age in Sydney and 40–69 years of age in New York (*14*)	
	35–39	0.52
	40–44	0.59
	45–49	0.66
	50–54	0.73
	55–59	0.80
	60–64	0.87
	65–69	0.94
γ	Age-specific rates of vaccine-modified smallpox for healthy vaccinated persons 35–69 years of age in Sydney and 40–69 years of age in New York (*14*,*15*)	
	35–39	0.13
	40–44	0.11
	45–49	0.09
	50–54	0.07
	55–59	0.05
	60–64	0.03
	65–69	0.01
β_1	Transmission parameter for hemorrhagic type (*14*)	0.625 (R_0 = 10), assuming cases are isolated from the first day of infectious period: 0.625/2 (R_0 = 5)
β_2	Transmission parameter for flat type (Assumed to be the same as the hemorrhagic smallpox)	0.625 (R_0 = 10), assuming cases are isolated from the first day of infectious period: 0.625/2 (R_0 = 5)
β_3	Transmission parameter for ordinary type (*19*)	0.497 (R_0 = 7.96) for the first 2 d of the infectious period and 0.497/2 (R_0 = 3.98) for the other 14 d for isolation
β_4	Transmission parameter for modified smallpox (Assumed to be 2/3 than the one for ordinary)	0.331 (R_0 = 5.3) for the first 3 d of the infectious period and 0.331/2 (R_0 = 2.65) for the other 13 d for isolation
$1/d_1$	Duration of latency + prodromal period (noninfectious) (*10*,*14*)	12 d + 3 d = 15 d
$1/d_3$	Duration of infectious period (*10*,*13*)	16 d
μ_1	Death rate for hemorrhagic smallpox (*15*)	0.9 for each age group
μ_2	Death rate for flat smallpox (*15*)	0.75 for each age group
μ_3	Death rate for ordinary smallpox for all age groups derived from linear interpolation through available data (*15*)	
	0–4	0.37
	5–9	0.18
	10–14	0.18
	15–19	0.15
	20–24	0.22
	25–29	0.29
	30–34	0.32
	35–39	0.35
	40–44	0.38
	45–49	0.40
	50–54	0.43
	55–59	0.46
	60–64	0.48
	65–69	0.51
	70–74	0.54
	75–79	0.56
	80–84	0.59
	≥85	0.62
μ_4	Death rate for vaccine-modified smallpox (*15*)	0 for each age group

*R_0, basic reproductive number; SEIR, susceptible, exposed, infected, recovered.

Technical Appendix Table 3. Distribution rates of hemorrhagic and flat smallpox for healthy unvaccinated infected persons, by age, New York, NY, USA, and Sydney, Australia

	Smallpox type	
Age, y	Hemorrhagic	Flat
0–4	0.012	0.08
5–9	0.007	0.045
10–14	0.026	0.026
15–19	0.049	0.035
20–24	0.085	0.043
25–29	0.122	0.054
30–34	0.132	0.065
35–39	0.141	0.075
40–44	0.151	0.086
45–49	0.161	0.97
50–54	0.171	0.108
55–59	0.180	0.118
60–64	0.190	0.129
65–69	0.200	0.14
70–74	0.210	0.151
75–79	0.220	0.162
80–84	0.229	0.172
≥85	0.239	0.183

Technical Appendix Table 4. Distribution rates of hemorrhagic, flat, and vaccine-modified smallpox for healthy vaccinated infected persons, by age, New York, NY, USA, and Sydney, Australia

	Age, y						
Smallpox type	35–39	40–44	45–49	50–54	55–59	60–64	65–69
Hemorrhagic	0.037	0.045	0.053	0.063	0.072	0.084	0.094
Flat	0.019	0.026	0.032	0.04	0.047	0.057	0.066
Vaccine-modified	0.19	0.18	0.17	0.16	0.15	0.14	0.13
